# Stage-Specific Sampling by Pattern Recognition Receptors during *Candida albicans* Phagocytosis

**DOI:** 10.1371/journal.ppat.1000218

**Published:** 2008-11-28

**Authors:** Sigrid E. M. Heinsbroek, Philip R. Taylor, Fernando O. Martinez, Luisa Martinez-Pomares, Gordon D. Brown, Siamon Gordon

**Affiliations:** 1 Sir William Dunn School of Pathology, University of Oxford, Oxford, United Kingdom; 2 Institute of Infectious Disease and Molecular Medicine, University of Cape Town, Cape Town, South Africa; David Geffen School of Medicine at University of California Los Angeles, United States of America

## Abstract

*Candida albicans* is a medically important pathogen, and recognition by innate immune cells is critical for its clearance. Although a number of pattern recognition receptors have been shown to be involved in recognition and phagocytosis of this fungus, the relative role of these receptors has not been formally examined. In this paper, we have investigated the contribution of the mannose receptor, Dectin-1, and complement receptor 3; and we have demonstrated that Dectin-1 is the main non-opsonic receptor involved in fungal uptake. However, both Dectin-1 and complement receptor 3 were found to accumulate at the site of uptake, while mannose receptor accumulated on *C. albicans* phagosomes at later stages. These results suggest a potential role for MR in phagosome sampling; and, accordingly, MR deficiency led to a reduction in TNF-α and MCP-1 production in response to *C. albicans* uptake. Our data suggest that pattern recognition receptors sample the fungal phagosome in a sequential fashion.

## Author Summary

Infection by *Candida albicans* has increased as a result of immunosuppression associated with AIDS and organ transplantation. We assessed the role of three pattern recognition receptors, namely Dectin-1 (a beta glucan receptor), the type 3 complement receptor (CR3), and the mannose receptor, in mediating uptake of this fungus. These receptors are known to recognize structures on the *C. albicans* cell wall, but their exact contribution to binding and uptake is still unclear. We show that only Dectin-1 plays a major role in binding and uptake of *C. albicans*. Furthermore, we are the first to find that these receptors sample the internalized particle in a sequential manner; intracellular mannose receptor is recruited later and is involved in secretion of immune modulators. These findings provide a better understanding of the innate immune mechanisms involved in protection against this medically important fungal pathogen.

## Introduction

*C. albicans* is an opportunistic pathogen, mostly confined to the gastrointestinal, genitourinary tracts and the skin. It can cause infections of mucosal tissues and is also able to invade systemically, especially in immunocompromised hosts [1]. Immune cells recognize *C. albicans* mainly through motifs on its cell wall which consists of approximately 90% carbohydrate, 5–10% protein and a small proportion of glycolipids [2]. The majority of the cell wall carbohydrates are β1,3- and β1,6-linked glucose polymers (β-glucans); it also contains a large amount of mannose polymers (mannans) covalently associated with protein, and a small amount of chitin [2]. The composition of the cell wall is dynamic and changes during cell growth and the transition of yeast to hyphal growth [3]. The cell wall is suggested to be layered and the inner layer adjacent to the plasma membrane is mainly composed of glucan and chitin that form a rigid structure giving the cell its morphology and protecting it from osmotic and other environmental factors [4]. The outer layer of the wall is primarily composed of mannans, the majority of which are covalently linked to the inner glucan layer [4]. The cell wall mannans and glucans can be recognised by pattern recognition receptors (PPR) of the immune system which then mediate subsequent binding, phagocytosis and stimulation of a pro-inflammatory response [5],[6].

Cells of the innate immune system express a range of PRR for fungal recognition that mediate the first encounter with *C. albicans*. These include various Toll-like receptors (TLR) that have been suggested to play a role in the cytokine response to fungal cells including TLR2, TLR4, TLR6 and TLR9 [7]–[11] and MyD88-dependent signalling may be required for resistance to *C. albicans* [12],[13]. A well characterised mechanism for yeast recognition is mediated via Dectin-1 where the subsequent inflammatory response involves collaboration between Dectin-1 and TLR2 [14],[15]. Dectin-1 recognises 1–3 linked β-glucans, mediating recognition and phagocytosis of a range of fungi including *C. albicans* [14], *Aspergillus fumigatus* [16], *Pneumocystis carinii* [17] and *Coccidioides posadasii* [18]. Other fungi are able to escape Dectin-1 recognition; for example, immune responses against *Histoplasma capsulatum* are suppressed by the masking of β-glucans with α-glucans [19]. A study by Gantner *et al.* showed that *C. albicans* binds Dectin-1 in a restricted pattern, concentrated in discrete subdomains on the yeast surface and particularly strong in the region between the parent cell and the mature bud, so-called bud and birth scars. By contrast, *C. albicans* filaments failed to bind soluble Dectin-1, not due to active inhibition, but rather a failure to provide access to the probe [20]. Besides mediating phagocytosis, dectin-1 also signals release of reactive oxygen, TNF-α, IL-2, IL-6, IL-10, and IL-23 [21],[22].

The mannan component of yeast cell walls has been suggested to be recognized by various receptors. One of these is the mannose receptor (MR). COS-cells were able to phagocytose *C. albicans* upon transfection with MR [23]. Studies using mannosyl inhibitors and cells overexpressing MR support a role for this receptor as phagocytic receptor [24],[25]. However, Le Cabec *et al.* suggested that whilst MR is able to bind ligands, it is unable to mediate phagocytosis upon binding [26]. Recognition of *C. albicans* N-linked mannosyl residues by the MR leads to TNF-α and IL-6 production [27], DC-SIGN on DCs and a mouse homologue SIGN-R1 have also been shown to recognise mannan structures [28],[29]. Dectin-2 has been shown to recognise mannan on *C. albicans*, primarily the hyphal form, inducing TNFα and IL-1Ra secretion upon recognition [30]–[32]. Signalling via Dectin-2 is dependent on an interaction with FcRγ [32]. The complement receptor 3 (CR3) lectin binding domain has also been shown to recognise *C. albicans* [33],[34], and it could bind both mannans and glucans [35]. Recently, it was shown that 1–6 linked β-glucans can be opsonised by C3b which then mediates opsonic phagocytosis via CR3 [36].

The particles a professional phagocyte encounters are likely to engage more than one phagocytic receptor, resulting in a tailored downstream signalling response to regulate phagosome maturation, changes in phagosomal pH, oxygen-dependent and -independent killing and cytokine secretion. Phagocytosis of *C. albicans* is more efficient when opsonised with serum; then both FcγR and CR3 mediate recognition of exposed antibody or iC3b components respectively [37],[38]. Furthermore, TLRs have also been shown to sample the phagosomal content and to regulate phagosome maturation and antigen presentation [39],[40]. Together, this variety of receptors greatly increases the complexity of the response to phagocytic stimuli, due to cross-talk and synergy of downstream signalling pathways. The mechanisms that enable Mφ to produce a response that is appropriate to the ingested particle are poorly understood [41],[42].

In this paper we determined the relative contribution of CR3, Dectin-1 and MR to the recognition of non-opsonised *C. albicans* and assessed their localisation during different stages of phagocytosis by Mφ. We show that CR3 and MR are not required for phagocytosis of *C. albicans* and consistent with other reports show that Dectin-1 is the main receptor involved in uptake of these particles. However, CR3 and Dectin-1 accumulate at the site of particle binding, suggesting other roles for CR3 during fungal recognition. These receptors largely disappear from the phagosome within 20 minutes of phagosome formation. MR, which is mainly localised intracellularly in the endosomal compartment, is not readily detectable in association with the particles during nascent phagosome formation, but accumulates at the phagosomes 20 minutes after initiation of phagocytosis. Although MR does not seem to be involved in uptake of *C. albicans* we show that it is required for cytokine production upon recognition. This is the first report to describe sequential sampling by pattern recognition receptors during phagocytosis.

## Results

### Receptor involvement in *C. albicans* phagocytosis

MR, Dectin-1, and CR3 have been suggested to mediate fungal phagocytosis [14],[23],[33],[34],[43]. To evaluate their relative contribution to non-opsonised *C. albicans* phagocytosis by macrophages (Mφ) we assessed their involvement using uptake assays. We chose to use thioglycollate-elicited macrophages because we wanted to examine dectin-1, MR and CR3 in the same system and these cells express all three receptors. In contrast, resident peritoneal do not express MR and alveolar macrophages do not express CR3 [44]. SIGN-R1 and Dectin-2 are not significantly expressed by thioglycollate elicited macrophages [29],[45]. Soluble mannans and β-glucans were used to block recognition of the main known pathogen associated patterns exposed on the surface of *C. albicans*. To investigate the involvement of pattern recognition receptors, conditions were kept free of opsonins by use of serum free medium; cells were also washed to remove potential locally secreted opsonins.

**Figure 1:**
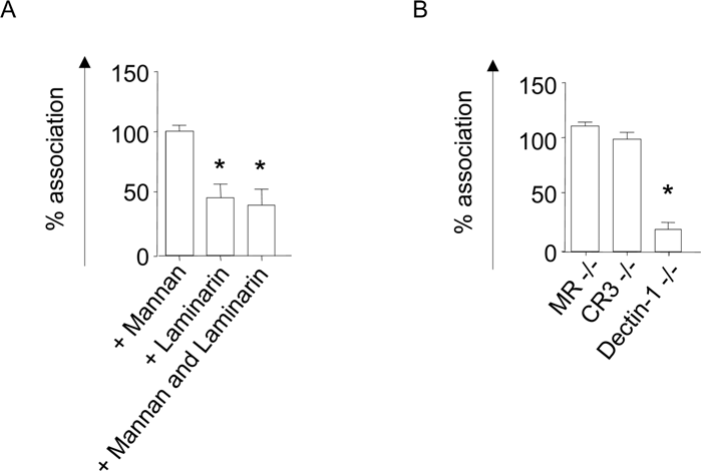
Assessment of Mφ receptor involvement in *C. albicans* binding and phagocytosis. (A) Association of *C. albicans* with thioglycollate elicited Mφ of BALB/c mice. Mφ were incubated with 100 µg/ml mannan, 100 µg/ml laminarin or both, for 30 minutes at 4°C, fluorescently labelled live *C. albicans* was added subsequently and preparations were incubated for 30 minutes at 37°C. Data are expressed as percentage association relative to untreated control cells. (B) Association of *C. albicans* with thioglycollate-elicited peritoneal MΦ of MR, CR3, or Dectin-1 deficient mice after incubation for 30 minutes at 37°C. Results are expressed as percentage association relative to untreated control cells. Data are representative of three independent experiments done in duplicate, error bars indicate the SD. A one-way ANOVA with Bonferroni multiple comparison test was used for statistical analysis. *, p<0.05.

We found that addition of mannan did not influence uptake of these particles, suggesting that mannan recognising receptors like MR are not involved in this process. Laminarin, a soluble β-glucan known to inhibit Dectin-1 [46], significantly reduced *C. albicans* uptake (Figure 1A). We next assessed *C. albicans* association using thioglycollate-elicited peritoneal MΦ isolated from mice deficient in MR, CR3 or Dectin-1. Mφ deficient in MR or CR3 did not show any impairment in phagocytosis of this yeast (Figure 1B). The role of Dectin-1 in recognition of *C. albicans* has been demonstrated using Mφ from Dectin-1-deficient mice [47] and we confirmed that in the absence of this receptor, association of these particles was reduced by 80% (Figure 1B). Residual association may be due to additional adhesion molecules known to be expressed by live *C. albicans*, or other receptors [48]–[50]. Together, these data suggest that Dectin-1 is the main pattern recognition receptor for uptake of non-opsonised *C. albicans* by thioglycollate-elicited peritoneal MΦ.

### Dectin-1 and CR3 both accumulate during phagocytosis of fungal particles

To assess the role of these receptors in particle recognition, we used confocal microscopy to analyse their localisation during binding and uptake. Since no good anti-Dectin-1 antibodies were available for staining fixed cells, we developed a new monoclonal antibody 7G7. 7G7 specifically recognised Dectin-1 (Figure S1A) and 7G7 staining of Dectin-1 on thioglycollate elicited MΦ showed that it was mainly expressed on the plasma membrane (Figure S1B).

**Figure 2:**
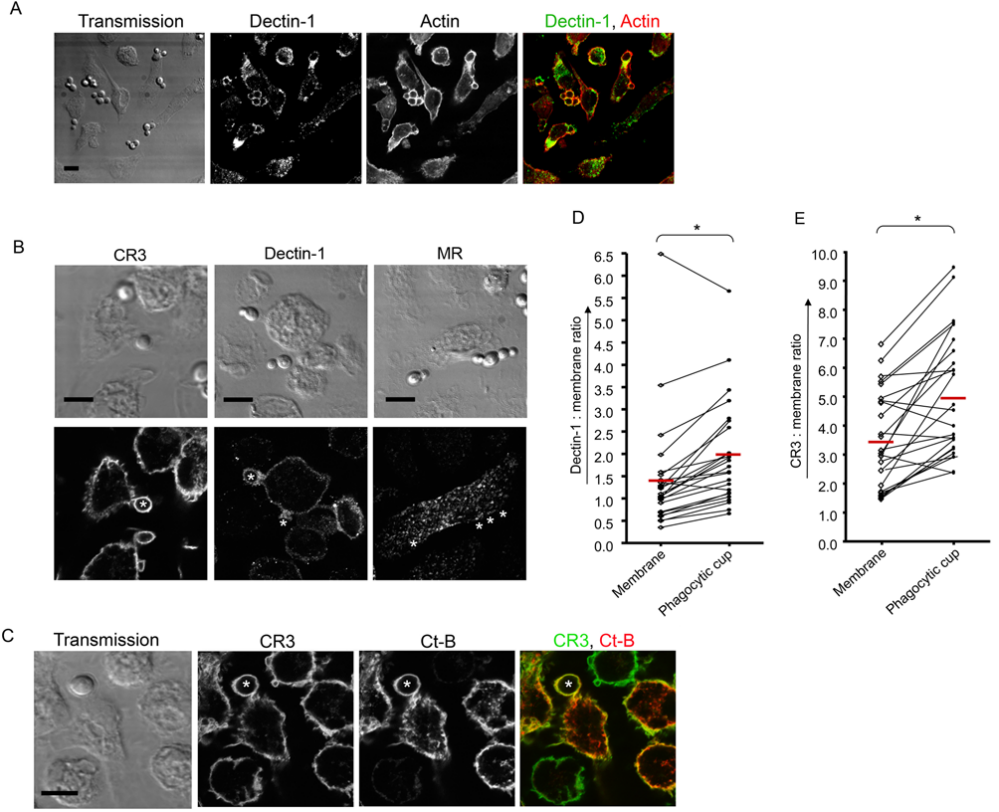
Dectin-1 and CR3 accumulate at the *C. albicans* phagocytic cup two minutes after onset of phagocytosis. (A) Thioglycollate-elicited peritoneal Mφ were challenged with *C. albicans*. Samples were fixed 2 minutes after initiation of phagocytosis and stained for actin (red) to localise phagocytic cups. Dectin-1 staining (green) shows receptor accumulation at the phagocytic cups. (B) Localisation of receptors 2 minutes after Mφ were challenged with *C. albicans*. Preparations were fixed and stained for CR3, Dectin-1, or MR. Top panel represents transmission images and the lower panel contains the corresponding fluorescent images. * indicates some of the particles. (C) A typical image for ratiometric analysis of a Mφ, two minutes after *C. albicans* phagocytosis. Thioglycollate-elicited peritoneal Mφ were labelled with membrane dye, either choleratoxin B or PKH26 (second panel), before *C. albicans* challenge, and stained for CR3 (first panel) after fixation. The combined colour image in the last panel shows CR3 in green and cholera toxin B in red. * indicates the particle. (D) Ratiometric data of Dectin-1 localisation. Cells were treated as described in C and 25 representative images were taken for each experiment. For each image receptor: Membrane ratios of mean intensities were calculated for the membrane around the particle and the plasma membrane by selecting three random regions in these areas. These data show the average ratio of the three regions at the membrane (◊) and the paired particle membrane (• ) from the same cell connected by a line. The sample mean is indicated with a red line. A paired *t*-test (two tailed) was used for statistical analysis.*, p<0.05. (E) Ratiometric data of CR3 as described in D. All images are representative of three independent experiments. Scale bars correspond to 10 microns.

To determine the localisation of MR, CR3 and Dectin-1 during phagocytosis, macrophages were stained for these receptors two minutes after onset of synchronised *C. albicans* phagocytosis. At this time phagocytic cups had formed, as could be seen from the accumulation of actin around the particles (Figure 2A). Association of *C. albicans*, mainly mediated by Dectin-1, was consistent with Dectin-1 localisation (Figure 2A and 2B). Surprisingly, CR3 also became associated with these fungal particles even though it had no detectable role in uptake (Figure 2B). MR which is mainly intracellular in location, did not show any association with the particles during this early stage of phagocytosis (Figure 2B).

Our results indicated that Dectin-1 and CR3 accumulate at the *C. albicans* phagosome, but this did not exclude the possibility that the receptors are enriched due to concentration of membrane around the particles. To address this further we measured receptor accumulation using ratiometric confocal microscopy. Besides receptor staining, PKH26 or Alexa 555-labelled cholera toxin B (Figure 2B) were used as general membrane dyes, to stain the plasma membrane uniformly. The fluorescence intensity ratio of the receptor and membrane staining at different cellular regions were used to assess receptor enrichment. The PKH26 dye contains a highly aliphatic reporter molecule that traps the probe once incorporated into the membrane, because of its inherent insolubility in an aqueous environment [51], and Alexa 555-labelled cholera toxin B subunit binds ganglioside GM1, localised to the Mφ plasma membrane. Ratiometric analysis showed that Dectin-1 (Figure 2D) and CR3 (Figure 2E) receptor levels at the *C. albicans* phagocytic cup increased significantly, with 33% and 42% respectively compared to the rest of the plasma membrane. Latex bead uptake, used as a negative control, did not show enrichment of CR3 during uptake (Figure S2). Taken together, these data confirm that both Dectin-1 and CR3 accumulate at the site of *C. albicans* phagocytosis, while MR does not.

### MR associates with fungal particles 20 minutes after phagocytosis

**Figure 3:**
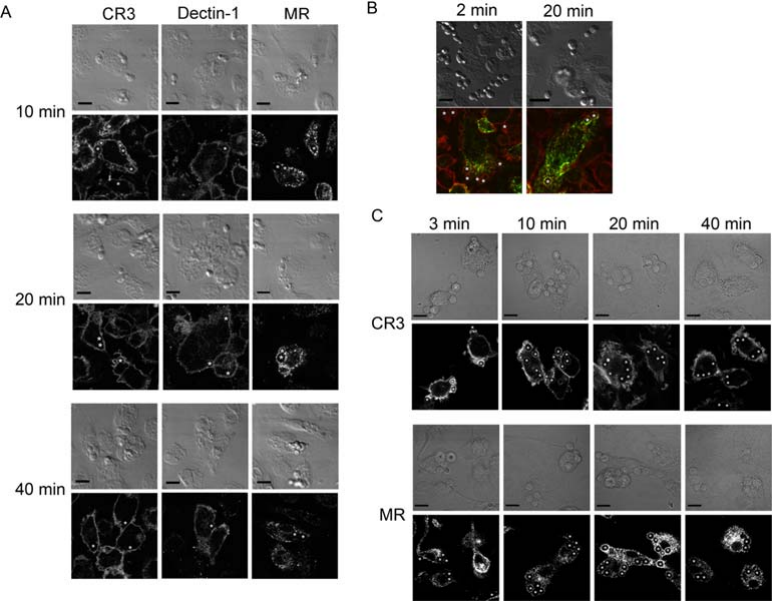
Macrophage receptor localisation at 10, 20, and 40 minutes after phagocytosis of *C. albicans*. Thioglycollate-elicited peritoneal Mφ were challenged with *C. albicans*. (A) Experiments were fixed after 10, 20, and 40 minutes and stained for CR3, Dectin-1 or MR. (B) Double staining of CR3 (red) and MR (green) of Mφ challenged with live *C. albicans*, 2 and 20 minutes after initiation of phagocytosis. (C) Human monocyte derived Mφ were challenged with *C. albicans*. Preparations were fixed after 3, 10, 20, and 40 minutes and stained for CR3, or MR. Top panels represent transmission images and the lower panels represent the corresponding fluorescent images.* indicates some of the particles. All images are representative of three independent experiments done in macrophages from different blood donors. Scale bars correspond to 10 microns.

To assess the dynamics of phagosomal association of the receptors, we next determined receptor localisation at later timepoints after phagosome formation. We found that the CR3 and Dectin-1 started to disappear from the phagosomes around 10 minutes after onset of phagocytosis (Figure 3A) and 20 minutes after initial binding these receptors had for the most part disappeared from the phagosomes, leaving only a few phagosomes stained for these receptors (Figure 3A). In contrast, the MR started to accumulate at the phagosomes at 10 minutes and this receptor had clearly localised at the phagosomes after 20 minutes (Figure 3A). The localisation of MR was transient since at 40 minutes phagosomes were largely negative for MR (Figure 3A). When cells were stained for both MR and CR3 we found that localisation of these receptors around the fungal particles was transient and stage specific with little colocalisation of these two receptors (Figure 3B). Similar transient localisation of CR3 followed by transient localisation of MR was also found during *C. albicans* phagocytosis in human monocyte derived macrophages, demonstrating that this phenomenon is not specific for murine macrophages (Figure 3C). Together these data show that *C. albicans* phagosomes associate with MR in a transitory manner while the phagosomes mature.

### Receptor dynamics are not specific for *C. albicans*

We next assessed if the receptor involvement and localisation during *C. albicans* phagocytosis is specific to this fungus, since it has been shown that *C. albicans* phagosomes mature faster than other phagosomes [52]. To determine this we looked at zymosan uptake. Zymosan is a crude β-glucan- and mannan-rich cell wall extract of *Saccharomyces cerevisiae* routinely used as a model for fungal particles [53]. We found that addition of mannan did not influence uptake of these particles while laminarin significantly reduced zymosan uptake (Figure S3A). Also macrophages deficient in MR or CR3 did not show any impairment in phagocytosis of this particle (Figure S3B). Mφ from Dectin-1-deficient mice showed a reduction in uptake of more that 95% (Figure S3B), suggesting that Dectin-1 is the major pattern recognition receptor for zymosan uptake by thioglycollate-elicited peritoneal Mφ.

**Figure 4:**
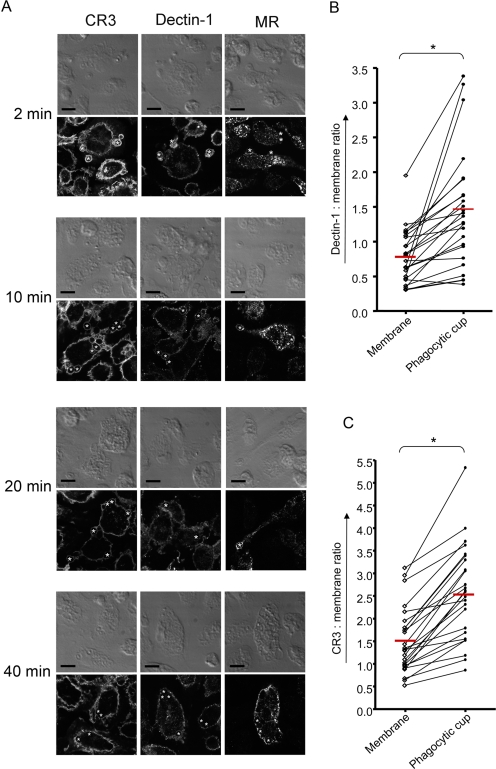
Receptor dynamics during phagocytosis of zymosan. (A) Thioglycollate-elicited peritoneal Mφ were challenged with zymosan. Preparations were fixed after 2,10, 20, and 40 minutes and stained for CR3, Dectin-1, or MR. Top panel represents transmission images and the lower panel contains the corresponding fluorescent images. * indicates some of the particles. All images are representative of three independent experiments. Scale bars correspond to 10 microns. (B) Ratiometric analysis of Dectin-1 localisation during zymosan phagocytosis. Thioglycollate elicited Mφ were stained with a membrane dye, either cholera toxin B or PKH26. MΦ were challenged with zymosan and stained for Dectin-1. 25 representative images were taken for each experiment. For each image receptor: Membrane ratios of mean intensities were calculated for the membrane around the particle and the plasma membrane by selecting three random regions in these areas. These data show the average ratio of the three regions at the membrane (◊) and the paired particle membrane (•) from the same cell connected by a line. The sample mean is indicated with a red line. A paired *t*-test (two tailed) was used for statistical analysis. *, p<0.05. C) Ratiometric data of CR3 as described in B.

Next, we assessed localisation and accumulation of these receptors during uptake. As with *C. albicans* uptake, Dectin-1 and CR3, but not the MR, accumulated at the phagocytic cup (Figure 4A). As before, the levels of Dectin-1 and CR3 were increased at the zymosan phagocytic cup compared to the rest of the membrane (Figure 4B and 4C). At later stages Dectin-1 and CR3 disappeared from the zymosan phagosomes while MR was clearly observed on phagosomes 20 minute post-uptake and at 40 minutes this receptor had also dissociated from the phagosomes (Figure 4A). These data suggest that such receptor dynamics during phagocytosis are not specific for *C. albicans*, but are a common feature for fungal particles.

### MR plays a role in cytokine response to fungal particles

**Figure 5:**
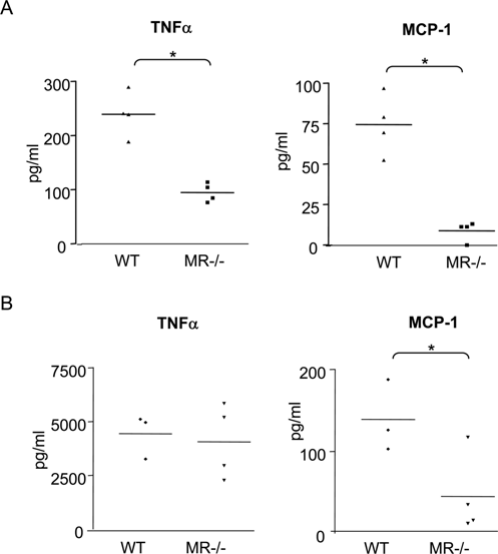
Cytokine production by MR−/− macrophages after phagocytosis of fungal particles. (A) Mφ were incubated with *C. albicans* for 30 minutes at 37°C. Unbound yeast were removed by washing and preparations were incubated at 37°C for 3.5 hours. Data show levels of TNF-α and MCP-1. (B) Cytokine levels after incubation with zymosan, as described in (A). Graphs represent pooled data of two independent experiments. A *t*-test was used for statistical analysis. *, p<0.05.

Although MR does not seem to play a crucial role in *C. albicans* uptake, it has previously been reported that *C. albicans* recognition by MR influences cytokine responses [27]. We assessed MCP-1 and TNFα production by MR deficient thioglycollate elicited macrophages upon stimulation with *C. albicans*, and found that after 4 hours MCP-1 and TNF-α production was reduced by 88% and 60%, respectively, compared to wild type cells (Figure 5A). We also assessed cytokine production upon stimulation with zymosan and found that while MCP-1 levels were reduced by 68% in MR-deficient cells, there was no significant difference in TNF-α production (Figure 5B). Together these data suggest that MR plays a role in cytokine responses towards fungal particles.

## Discussion

**Figure 6:**
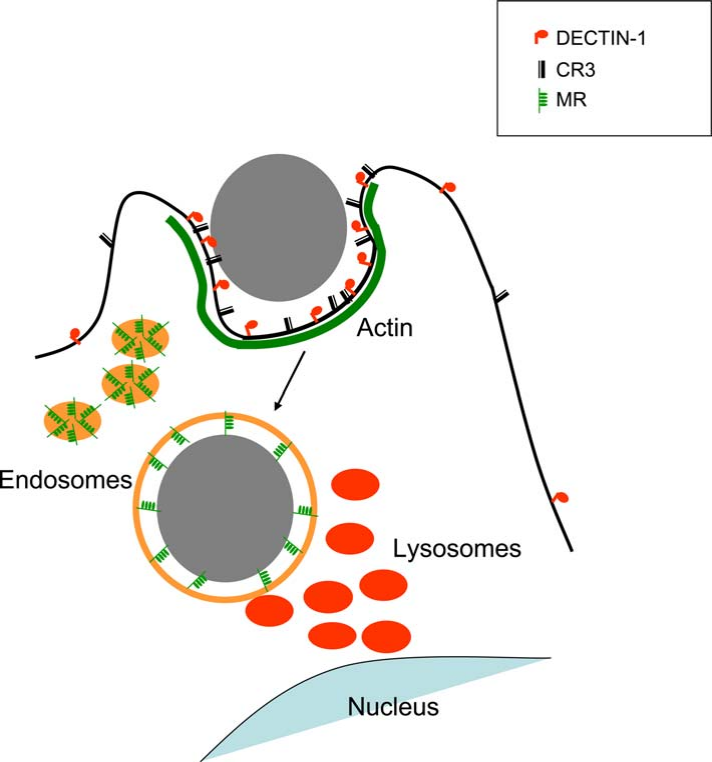
Proposed model for sequential receptor localisation during phagocytosis of fungal particles. Dectin-1 is the main receptor for fungal uptake; however, CR3 also accumulates at the site of phagocytosis possibly collaborating with Dectin-1. Once a phagosome is formed, these receptors disappear from the phagosome; some are degraded and others recycled to the plasma membrane. At this stage, MR located within the endosomes become incorporated in the phagosomal membrane, where they are able to sample the phagosome. While the phagosome matures, MRs also leave the phagosome.

The *C. albicans* cell wall is composed of layers containing different ligands [4]. Recognition of these ligands by PRR can mediate phagocytosis and regulate immune responses. In this study we analysed the role of the MR, Dectin-1 and CR3 in phagocytosis of non-opsonised *C. albicans*. Taken together, our observations suggest a model of sequential localisation of pattern recognition receptors during phagocytosis of fungal particles. We showed that Dectin-1, but not CR3 or MR, is a major receptor for uptake of *C. albicans* under opsonin free conditions. However, during phagosome formation CR3 is also recruited to the particle, possibly contributing to phagosome maturation. Later during phagosome maturation these receptors disappear from the phagosome. Outer layers of the phagosomal content will be degraded while the phagosome matures and other pattern recognition receptors like TLRs and MR accumulate around the maturing phagosome where they can sample ligands exposed during particle degradation, thus modulating cytokine responses (Figure 6). Sequential localisation of proteins during endocytosis and endosome maturation was recently shown to provide access to distinct signalling pathways [54].

Before the discovery of Dectin-1 as a β-glucan receptor, β-glucan recognition was suggested to be mediated by CR3. Studies using anti-CR3 antibodies and glucan containing carbohydrates to block opsonin independent recognition and phagocytosis of zymosan and *C. albicans* supported this hypothesis [33],[35],[43],[55],[56]. However, because of colocalisation of Dectin-1 and CR3 at the phagocytic synapse (Figure 2), it is possible that studies where anti-CR3 antibodies were used to block zymosan and *C. albicans* binding, Dectin-1 was sterically hindered by antibodies bound to CR3. Using CR3-deficient Mφ, we have shown that CR3 does not play an essential role in the association and phagocytosis of zymosan or *C. albicans* (Figure 1 and Figure S3). Nevertheless, like Dectin-1, CR3 did accumulate at the phagocytic synapse (Figure 2). Our ratiometric data assessed Dectin-1 and CR3 levels at the site of phagocytosis compared to the rest of the plasma membrane, showing that these receptors become enriched around the fungal particles (Figure 2). It is possible that CR3 and Dectin-1 interact with each other during the uptake of yeast particles, since CR3 has been shown to collaborate with other receptors for phagocytosis and adhesion [57]–[59].

Our experiments show that Dectin-1 is the main receptor involved in phagocytosis of unopsonised *C. albicans*, confirming several reports which show a role for Dectin-1 in *C. albicans* phagocytosis [14],[46],[47]. However, one paper has shown contradicting data on the recognition of *C. albicans* by macrophages from Dectin-1 deficient mice [60], differences in fungal strain and mouse genetic background have been suggested as possible causes for this difference [61]. In this study we used the same *C. albicans* strain also with thioglycollate elicited macrophages as described by Saijo *et al.* [60] and in our hands Dectin-1 is the main receptor for phagocytosis of *C. albicans* ATCC18804 strain. This suggests that the difference in mouse strain cause differences in Dectin-1 involvement; we have previously shown that different mouse strains can express different isoforms which influence ligand binding and subsequent immune responses [62]. It is not clear if other differences in the methods used by Saijo *et al.* could account for their differences from the other published reports.

Dectin-1 deficient macrophages show a residual uptake of 20%, this may be due to additional receptors. SIGN-R1 and Dectin-2 are not expressed by thioglycollate-elicited macrophages [29],[45]. It is possible that TLR2 contribute to the phagocytic process. TLR2 is known to collaborate with Dectin-1 for inflammatory responses upon *C. albicans* recognition and has been shown to influence uptake of the fungus *Aspergillus fumigatus* [14],[15],[63]. Furthermore, Blander *et al.* showed that TLR2−/−TLR4−/− deficient cells have reduced bacterial uptake [39]. On the other hand, Gantner *et al.* showed that macrophages deficient in TLR2 or Myd88 have no reduced phagocytosis of zymosan [15]. Together, it is unclear which receptors or adhesins mediate the remaining 20% of dectin-1-independent *C. albicans* association with macrophages.

Different experiments have led to the suggestion that MR could be involved in phagocytosis of mannan exposing particles; soluble MR was shown to bind *C. albicans* [25] and subsequent inhibition of Mφ recognition with mannan blocked the interaction with *C. albicans* [25] and other mannan exposing particles [64]–[68]. Also, COS cells over-expressing MR have been shown to phagocytose zymosan [23]. We were unable to demonstrate a role for MR in the binding and phagocytosis of *C. albicans* or zymosan by Mφ (Figure 1), which is consistent with previous reports [26],[69]. We observed that MR was not located around the phagocytosed particles during binding or initial phagocytosis (Figure 2). Although MR is able to recognise yeast particles [25], the level of its expression at the surface of primary Mφ may not be sufficient to initiate binding and/or phagocytosis. Other mannan recognising receptors have been suggested to be involved in phagocytosis; these include DC-SIGN [28] and the mouse homologue SIGN-R1 [29]. Interactions of these receptors are also inhibited with mannan. SIGN-R1 was not expressed on the thioglycollate elicited macrophages used in our experiments [29].

Previous work showed that MR is involved in initiating cytokine responses upon *C. albicans* mannan recognition [27],[70], and the receptor has also been implicated in phagocytosis of this yeast [23], [25], [64]–[68]. We showed that MR became enriched around the phagosome at later stages of phagosome maturation (Figure 3). This suggests that MR is appropriately positioned to recognise ligand intracellularly, directly or after further processing. Indeed, MR-deficient macrophages produce lower levels of TNF-α and MCP-1 in response to *C. albicans* (Figure 5). This is the first report showing a role for MR in MCP-1 production by macrophages. MR deficient macrophages did not show reduced TNF-α levels upon zymosan uptake, this could be explained by the large amount of TNF-α produced upon zymosan recognition which may mask MR influences on TNF-α levels. Intracellular compartments have been proposed to be the main site for TLR-mediated sampling of microbial components [71],[72], suggesting that the phagosome is a likely place for PRR to recognise ligands and mediate immune responses. This is the first report showing that MR also samples the maturing *C. albicans* phagosome, where it influences the cytokine response.

In summary, we have observed sequential association of macrophage pattern recognition receptors with the phagosome during its maturation. The timing of this association was receptor specific and appeared to reflect the natural compartmentalisation of the receptor itself. These studies provide new insights into the processes by which surface and predominantly intracellular receptors sample the phagosome. Taken altogether this suggests that pattern recognition receptors sample ligands both during phagocytosis and phagosome maturation to tailor a response specific for the internalised particle.

## Materials and Methods

### Mouse macrophage cultures

Mice used in this study (6 BALB/c, 35 C57BL/6, 10 C57BL/6.MR^−/−^ [73], 6 C57BL/6.CD11b^−/−^ [74] and 7 129/Sv×C57BL/6 Dectin-1^−/−^ and 6 129/Sv×C57BL/6 controls [47]) were from the Sir William Dunn School of Pathology (University of Oxford) breeding colonies, sex-matched and between 8 and 12 weeks of age at the time of study. Animals were kept and handled in accordance with institutional guidelines. Thioglycollate-elicited peritoneal Mφ were isolated 4 days after intraperitoneal injection of 1 ml 4% (w/v) Brewer's thioglycollate medium (BD). Primary Mφ were cultured overnight in serum-free defined medium, Optimem-I (Invitrogen) with 50 IU/ml Penicillin, 50 µl/ml streptomycin and 2 mM L-glutamine (Optimem medium) at 37°C in 5% CO_2_. For binding studies, 2.5×10^5^ cells were plated in 24 well tissue culture plates and cultured overnight. For confocal studies, 2.5×10^5^ cells were cultured overnight on acid washed 13 mm diameter glass coverslips in 24 well tissue culture plates. After 1 to 2 hours, non-adherent cells were removed by washing three times with medium.

### Human macrophage cultures

Human monocytes were obtained from normal blood donor buffy coats by two step gradient centrifugation followed by an additional step using the Monocyte Isolation Kit II (Miltenyi Biotec; Bergisch Gladbach, Germany) as described previously [75]. Macrophages were obtained by culturing monocytes (98% CD14^+^, 13% CD16^+^) for 7 days in X-VIVO 10 (Cambrex) supplemented with 1% autologous serum. Cells were cultured in Lumox hydrophobic dishes (Sigma) until day 7, with a partial medium change at day 3. On day 7 cells were detached with cold 10 mM EDTA and transferred into glass slides.

### Antibodies

The following primary antibodies were used for confocal microscopy experiments: rat IgG1 anti-Dectin-1 (7G7), biotinylated rat IgG1 isotype control (BD Pharmingen)), rat IgG2a anti mannose receptor ( clone 5D3, Serotec), rat IgG2b anti-CR3 (clone 5C6, Serotec), biotinylated anti-CR3 (clone 5C6) , fluorescein isothiocyanate–conjugated 7/4 (antibody to neutrophils and monocytes), phycoerythrin-conjugated anti-Ly-6G (clone 1A8) and rat IgG2b and IgG2a isotype controls (produced in house). Cy3 labelled streptavidin (Jackson ImmunoResearch) and Alexa 488 labelled goat anti-rat IgG (Molecular Probes) were used as secondary antibody.

### Characterisation of 7G7 antibody

The antibody 7G7 (rat IgG1) was produced as described [46]. NIH3T3, a fibroblast cell-line overexpressing HA-tagged Dectin-1, was used to confirm the specificity for Dectin-1 by confocal microscopy and FACS analysis. The NIH3T3 cells were cultured in RPMI1640 (Gibco) supplemented with 10% heat inactivated FCS, 100 IU/ml Penicillin G (Gibco), 0.1 mg/ml streptomycin (Gibco) and 2 mM glutamine (Gibco) (R10 medium). G418 (Sigma), 250 µg/ml, was added as selective agent for the transfected cells.

### *Candida albicans* culture

Stock cultures of *C. albicans* (ATCC 18804) were maintained on Sabouraud's dextrose agar (Difco) at 4°C. For experiments, *C. albicans* was grown in 10 ml Sabouraud's dextrose broth (Difco) in a shaking incubator at 30°C for 24 hours, to obtain a stationary phase culture.

### Phagocytosis assays

FITC-labelled zymosan was from Molecular Probes. Live *C. albicans* were fluorescently labelled by incubation with 20 µM FUN1 (Molecular Probes) as described previously [76]. Binding experiments were performed as described [62]. Briefly, Mφ were plated at 2.5×10^5^ cells/well in 24-well plates in the appropriate medium and cultured for 12 hours. Cells were cooled to 4°C and washed three times with pre-chilled medium to remove potential opsonins. To block receptor binding, 100 µg/ml mannan (Sigma) and/or laminarin (Sigma) were added in medium for 30 minutes at 4°C, prior to adding the particles. Fluorescently labelled particles were added to Mφ at a 20∶1 ratio and cells were incubated for 1 hour at 4°C or 30 minutes at 37°C. After incubation, unbound particles were removed by washing 4 times with pre-chilled PBS. The amount of *C. albicans* or zymosan associated with the cells was quantified after lysis with 3% w/v Triton X-100 solution, pH 8, using a fluoroscan II fluorometer (Titertek, Huntsville, Alabama, USA) at excitation/emission of 485/538 nm. Data were analysed for statistically significant differences using One-way ANOVA with Bonferroni multiple comparison test.

### Confocal immunofluorescence studies

Non-fluorescent zymosan (Invitrogen), 6.0 micron polystyrene polybead (Polysciences Inc., Warrington, Pennsylvania, United States of America) and live *C. albicans* were used for these studies. Mφ were cooled to 4°C and washed three times with pre-chilled medium. Non-fluorescent particles were added to the Mφ at a Mφ∶particle ratio of 1∶5, and incubated for 1 hour at 4°C. Unbound particles were removed by washing 3 times with pre-chilled medium. The cells were then incubated at 37°C for different periods. Cells were fixed with 4% paraformaldehyde (Sigma) in PBS or 1% formaldehyde (Sigma) in PBS for 15 minutes at 4°C. Cells were permeabilised in buffer containing 0.25% Saponin (Sigma), 1% BSA (Sigma), 1% heat inactivated goat serum (Sigma) and 1% heat inactivated rabbit serum (Sigma) in PBS (permeabilisation buffer) at room temperature for 30 minutes. Primary antibodies were added at a concentration of 10 µg/ml in permeabilisation buffer for 1 hour at room temperature. Cells were washed three times prior to incubation with the secondary antibody in the same buffer. After two washes with buffer and two washes with PBS, coverslips were mounted on glass microscopy slides with DakoCytomation Fluorescent Mounting Medium (DakoCytomation). Double staining for CR3 and MR was done in the buffers described above, as follows: 5D3 was added first after which an anti-rat secondary reagent was used. This was followed with biotinylated 5C6 and incubation with Cy3 conjugated streptavidin. FITC-phalloidin (Sigma) was added at a concentration of 10 µg/ml in permeabilisation buffer for 1 hour at room temperature. Experiments were analysed using a confocal microscope (Radiance 2000, Biorad) and representative images obtained using lasersharp software, with the pinhole set to the optimal size. Images were processed using Adobe Photoshop version 6.

### Ratiometric assays

For ratiometric analysis, Mφ plasma membranes were stained with 10 µg/ml Alexa fluor 555 conjugated cholera toxin B (Invitrogen) for 2–5 minutes at 37°C, or with PKH26 (Sigma) according to the manufacturer's protocols. Vybrant DiI (Invitrogen) and FM4-64FX membrane dye (Invitrogen) were also used according to the manufacturer's protocol, however the quality of staining was not adequate for our experiments. Following staining, preparations were treated as described above for confocal immunofluorescence studies. In brief, cells were cooled and washed, particles added and preparations kept at 4°C for 1 hour to allow binding of the particles. Unbound particles were removed by washing and cells incubated at 37°C for two minutes. Experiments were fixed on ice and stained as described above. The staining buffer for these experiments did not contain saponin, reducing membrane permeabilisation as much as possible to prevent loss of membrane staining.

For each experiment, 25 representative images were collected on a Radiance 2000 (Biorad) confocal microscope, using a 60× PlanApo oil immersion objective (NA 1.4). The software used for collecting images was Bio-Rad Lasersharp 2000 (v. 5.0). Kalman-filtered images were collected with an optimal iris aperture and the minimum laser power that fitted the whole grey scale. To prevent bleedthrough between channels, sequential series were obtained by alternating the different channels.

Fluorochrome intensity measurements were obtained for each image using MetaMorph. The mean intensity of each fluorochome was measured at three regions (regions 1–3) around the particle and three regions elsewhere on the plasma membrane (regions 4–6). The regions were standard 15*15 pixel squares. These data were exported to Excel where the receptor to membrane ratio was calculated as described below and plotted using GraphPad Prism.

The following calculation was used to assess the receptor intensity at the membrane and at the site of phagosome formation:
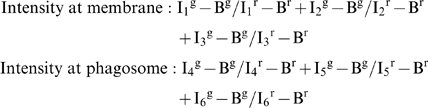
Where 1, 2 and 3 are regions from the phagosome and 4,5 and 6 are regions from the plasma membrane, I_j_^g^ is the intensity of green fluorescence (receptor) in region R_j_ and similarly I_j_^r^ for red fluorescence (membrane) in region R_j_. B^g^,B^r^ are the background intensity of green and red respectively.

The average receptor: membrane ratio was measured for three regions selected randomly at the Mφ plasma membrane and for another three regions selected randomly at the site of particle entry. Since both measured variables (plasma and phagosomal membrane staining) were derived from the same cell, receptor fluorescent intensity at the membrane and the phagosome was expressed for each cell as paired data. A paired *t*-test (two tailed) was used for statistical analysis. The sample mean was indicated in red.

### Cytokine assay

Mφ were plated at 2.5×10^5^ cells/well in 24-well plates in the appropriate medium and cultured for 12 hours. Cells were cooled to 4°C and washed three times with pre-chilled medium. Live *C.albicans* were added to Mφ at a 20∶1 ratio and cells were incubated for 30 minutes at 37°C. Unbound particles were removed by washing 3 times with PBS. 300 ul medium were added to the cells, which were incubated for 3.5 hours at 37°C. TNF-α, IL-6, MCP-1 and IL-10 were assessed in supernatants using BD Cytometric Bead Array (BD Biosciences). IL-6 and IL-10 levels were below detection limit of 20 pg/ml. A *t*-test was used for statistical analysis.

### Accession numbers

*Mus musculus* Dectin-1/Clec7a = Gene ID56644

  MR/mrc1 = Gene ID17533

  CD11b/Itgam = Gene ID16409

*Homo sapiens* Dectin-1/Clec7a = *ID*64581

  MR/mrc1 = ID4360

  CD11b/Itgam = ID120980

## Supporting Information

Figure S1 Characterisation of 7G7 for immunofluorescent staining. A) Representative FACS profiles of mouse bone marrow demonstrating 7G7 specificity for Dectin-1. Left plot shows gating on Ly-6Ghi7/4hi neutrophils (Neu) and monocytes (Mo), for analysis of dectin-1 specificity; dectin-1-wild-type (dectin-1-WT; middle) and dectin-1-knockout mice (dectin-1-KO; right) were stained with 7G7. B) Thioglycollate-elicited peritoneal Mφ were fixed and stained with Dectin-1 and matching isotype control antibodies. Images are representative for three independent experiments.
(1.29 MB PDF)

Figure S2 Ratiometric analysis of CR3 accumulation during latex bead uptake. Ratiometric analysis of CR3 localisation during latex bead phagocytosis. Thioglycollate elicited Mφ were stained with a membrane dye, either cholera toxin B or PKH26. MΦ were challenged with latex beads and stained for CR3. 25 representative images were taken for each experiment. For each image, receptor: membrane ratios of mean intensities were calculated for the membrane around the particle and the plasma membrane by selecting three random regions in these areas. These data show the average ratio of the three regions at the membrane (◊) and the paired particle membrane (♦) from the same cell connected by a line. The sample mean is indicated with a red line. A paired t-test (two tailed) was used for statistical analysis.*, p<0.05.
(0.01 MB PDF)

Figure S3 Assessment of macrophage receptor involvement in zymosan binding and phagocytosis. A) Association of zymosan with thioglycollate elicited MΦ of BALB/c mice. Mφ were incubated with 100 µg/ml mannan, 100 µg/ml laminarin or both for 30 minutes at 4°C, fluorescently labelled zymosan was added subsequently and preparations were incubated for 30 minutes at 37°C. Data are expressed as percentage association relative to untreated control cells. B) Association of zymosan with thioglycollate-elicited peritoneal Mφ of MR, CR3 or Dectin-1 deficient mice after incubation for 30 minutes at 37°C. These data are representative of three independent experiments done in duplicate, error bars indicate the SD. *, p<0.05. Data are expressed as percentage association relative to untreated control cells.
(0.01 MB PDF)
