# Supplementary figures and images for: Stage-Specific Sampling by Pattern Recognition Receptors during Candida albicans Phagocytosis
Source: PLoS Pathog. 2008 Nov 28;4(11):e1000218. doi: 10.1371/journal.ppat.1000218 (PMC2583056; doi:10.1371/journal.ppat.1000218)

A

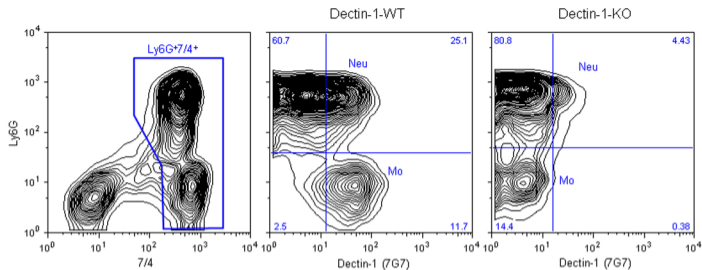

B

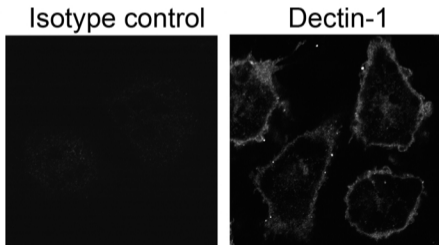

Supplement: Figure S1 — Characterisation of 7G7 for immunofluorescent staining. A) Representative FACS profiles of mouse bone marrow demonstrating 7G7 specificity for Dectin-1. Left plot shows gating on Ly-6Ghi7/4hi neutrophils (Neu) and monocytes (Mo), for analysis of dectin-1 specificity; dectin-1-wild-type (dectin-1-WT; middle) and dectin-1-knockout mice (dectin-1-KO; right) were stained with 7G7. B) Thioglycollate-elicited peritoneal Mφ were fixed and stained with Dectin-1 and matching isotype control antibodies. Images are representative for three independent experiments. (1.29 MB PDF) [file ppat.1000218.s001.pdf]

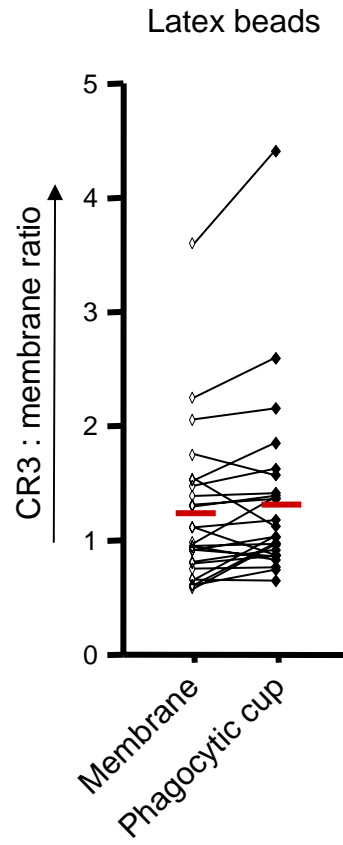

Supplement: Figure S2 — Ratiometric analysis of CR3 accumulation during latex bead uptake. Ratiometric analysis of CR3 localisation during latex bead phagocytosis. Thioglycollate elicited Mφ were stained with a membrane dye, either cholera toxin B or PKH26. MΦ were challenged with latex beads and stained for CR3. 25 representative images were taken for each experiment. For each image, receptor: membrane ratios of mean intensities were calculated for the membrane around the particle and the plasma membrane by selecting three random regions in these areas. These data show the average ratio of the three regions at the membrane (◊) and the paired particle membrane (♦) from the same cell connected by a line. The sample mean is indicated with a red line. A paired t-test (two tailed) was used for statistical analysis.*, p<0.05. (0.01 MB PDF) [file ppat.1000218.s002.pdf]

A

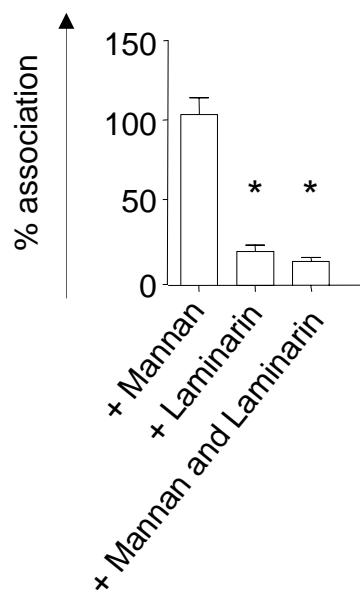

B

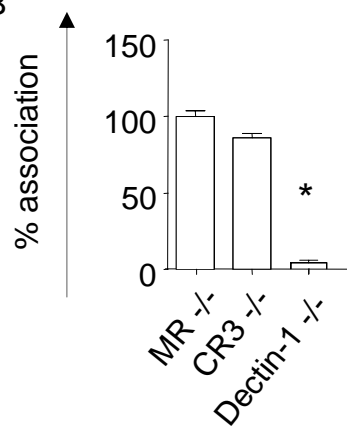

Supplement: Figure S3 — Assessment of macrophage receptor involvement in zymosan binding and phagocytosis. A) Association of zymosan with thioglycollate elicited MΦ of BALB/c mice. Mφ were incubated with 100 µg/ml mannan, 100 µg/ml laminarin or both for 30 minutes at 4°C, fluorescently labelled zymosan was added subsequently and preparations were incubated for 30 minutes at 37°C. Data are expressed as percentage association relative to untreated control cells. B) Association of zymosan with thioglycollate-elicited peritoneal Mφ of MR, CR3 or Dectin-1 deficient mice after incubation for 30 minutes at 37°C. These data are representative of three independent experiments done in duplicate, error bars indicate the SD. *, p<0.05. Data are expressed as percentage association relative to untreated control cells. (0.01 MB PDF) [file ppat.1000218.s003.pdf]
